# Rutin alleviates diabetic cardiomyopathy in a rat model of type 2 diabetes

**DOI:** 10.3892/etm.2014.2090

**Published:** 2014-11-26

**Authors:** YONG-BIN WANG, ZHI-MING GE, WEI-QIANG KANG, ZHE-XUN LIAN, JIAN YAO, CHANG-YONG ZHOU

**Affiliations:** 1 Department of Emergency Medicine, The Affiliated Hospital of Qingdao University, Qingdao, Shandong 266003, P.R. China; 2 Department of Cardiology, Qilu Hospital of Shandong University, Jinan, Shandong 250014, P.R. China; 3 Qingdao Municipal Hospital, Qingdao, Shandong 266011, P.R. China; 4 Department of Cardiology, The Affiliated Hospital of Qingdao University, Qingdao, Shandong 266003, P.R. China

**Keywords:** diabetic cardiomyopathy, rutin, diabetic rats, streptozocin, antioxidants

## Abstract

Diabetic cardiomyopathy (DCM), an independent coronary heart disease that develops in diabetic individuals, is characterized by changes in the myocardial structure and function. The aim of the present study was to investigate the protective effect of rutin on DCM in a streptozotocin-induced diabetic rat model. Rutin was orally administrated at a dose of 8 mg/kg body weight. Metabolic profiles, myocardial enzymes and oxidative stress were examined by biochemical tests. The expression levels of cellular proteins associated with apoptosis were measured by western blot analysis, while the levels of inflammatory factors were assessed by immunohistochemical analyses. Rats with DCM exhibited an abnormal metabolic profile, aberrant myocardial enzymes, elevation of oxidative stress markers, increased levels of inflammatory factors and enhanced apoptotic cell death. Notably, rutin was shown to protect and improve myocardial dysfunction, oxidative stress, apoptosis and inflammation in the hearts of the diabetic rats. In conclusion, these results indicated that rutin may have great therapeutic potential in the treatment of DCM, and possibly other cardiovascular disorders, by preventing oxidative stress, inflammation and cell death. However, further detailed studies are required to reveal the exact mechanisms underlying the protective effect of rutin.

## Introduction

Diabetes mellitus (DM) is accompanied by a number of complications due to the abnormal control of glycometabolism and lipid metabolism. Diabetic cardiomyopathy (DCM), a condition observed in diabetic individuals, is characterized by changes to the myocardial structure and function, independent of coronary artery disease and systemic hypertension (1,2). An increase in the levels of blood lipoproteins and free fatty acids facilitates the development of cardiovascular diseases, including hyperlipidemia and coronary artery disease, which can lead to further complications, such as retinopathy, nephropathy, neurosis, nephrotoxicity and hyperglycemia-induced coma (3). However, the development of DCM remains poorly understood and the underlying mechanisms have not yet been clearly elucidated. Diabetic complications are generally considered to be the result of oxidative stress (4), the excessive production of reactive oxygen species (ROS) and the aberration of the antioxidant system (5). In addition, diabetic complications are interrelated with the inflammatory response, and have been shown to be accelerated under a hyperglycemic state for the production of acute response factors in fat cells (6–8).

Rutin is a phenolic compound and flavonoid glycoside that is found in flowers and fruits as a major source. Rutin can be broadly extracted from nature sources, including buckwheat, oranges, grapes, lemons, limes, peaches and berries (9,10). The compound has been reported to possess dynamic pharmacological functions, including antioxidant, antibactericidal, antiviral (11,12), antitumor (13), anti-inflammatory (14), myocardial protection (15) and hepatoprotective (16) effects. In addition, previous studies have demonstrated the efficiency of the pharmacological functions of rutin as an antioxidant (11,17,18).

In the present study, considering the potential therapeutic properties of rutin, the aim was to investigate the protective effects of rutin on DCM and its involvement in the alterations of cardiac function and associated mechanisms in a rat model of DM.

## Materials and methods

### Experimental animals

Two-month-old male Wistar rats were procured from the Chinese People’s Liberation Army Military Academy of Medical Sciences Animal Experiment Center (Beijing, China). In total, 24 male Wistar rats (weight, 70–90 g) were used for the experiment. The animals were maintained with good ventilation and a 12-h light/dark cycle. Prior to the experiments, the animals were provided with food and water *ad libitum*. The animals were treated in accordance with the Guide for the Care and Use of Laboratory Animals published by the National Institutes of Health (NIH Publication no. 85-23, revised 1996). All experiments were approved by Institutional Animal Care and Use Committee of the Affiliated Hospital of Qingdao University (Qingdao, China).

### DM induction and rutin administration

To induce DM, the rats were fasted for 12 h, after which 65 mg/kg streptozotocin (STZ) dissolved in 0.1 M citrate buffer (pH 4.5) was intraperitoneally administered. The rats were fasted again for 12 h. At day 6 following STZ administration, the level of blood glucose was measured by collecting whole blood from the tail vein. Subsequently, the rats that had a blood glucose level of >350 mg/dl were screened for further experiments. The blood glucose level was measured using a glucometer (Accu-Chek Go model GS; Roche Diagnostics GmbH, Mannheim, Germany).

For the experiments, the rats were divided into three groups, which included the normal group (normal, n=8), STZ-induced DM group (DM, n=8) and rutin-treated DM group (DM + rutin, n=8). For the DM + rutin group, 8 mg/kg rutin dissolved in soybean oil was orally administered at the same time every day for one week following the induction of DM.

### Hematological analysis

At 72 h following the STZ injection, blood glucose levels were measured using a glucometer (Changsha Sinocare Inc., Changsha, China), following tail vein puncture blood sampling. Serum triglyceride (TG) and total cholesterol (TC) levels were determined using an auto-biochemical analysis system (AU2700; Olympus, Tokyo, Japan). The body weight was recorded every day for one week. After 12 days of rutin treatment, the experimental animals were euthanized by CO_2_ inhalation.

### Measurement of serum myocardial enzymes

Blood samples were collected from the abdominal artery and the serum was separated by centrifugation at 1,600 × g for 10min at 4°C. The levels of creatine kinase-MB (CK-MB), lactate dehydrogenase (LDH) and aspartate aminotransferase (AST) were determined using an auto-biochemical analysis system (AU2700; Olympus).

### Estimation of the superoxide dismutase (SOD) activity and malondialdehyde (MDA) level

Heart tissue samples were weighed and homogenized (1:10, w/v) in 50 mmol/l phosphate buffer (pH 7.4). The SOD activity and MDA level were measured using the appropriate detection kits A001-4 for SOD and A003-1 for MDA purchased from Nanjing Jiancheng Bioengineering Institute (Nanjing, China).

### Immunohistochemical staining

Paraffin-embedded sections underwent immunohistochemistry using a microwave-based antigen retrieval method. The sections were incubated with primary rabbit polyclonal anti-tumor necrosis factor-α (TNF-α; Abcam, Cambridge, MA, USA; #ab9635; dilution: 1 μg/ml) and anti-interleukin-6 (IL-6; Abcam; #ab6672; 1:500) antibodies overnight, and subsequently with a corresponding biotinylated anti-rabbit (#7074) and anti-mouse IgG (#7076) secondary antibody (Cell Signaling Technology, Inc., Danvers, MA, USA) for 30 min at 37°C. Negative controls were performed with the omission of the primary antibody. The results were viewed under a confocal FV1000 SPD laser-scanning microscope (Olympus).

### Western blot analysis

Frozen left ventricular tissue samples were homogenized in ice-cold lysis buffer [20 mM Tris (pH 7.5), 150 mM NaCl, 1 mM EDTA, 1 mM EGTA, 1% Triton X-100, 2.5 mM sodium pyrophosphate, 1 mM β-glycerolphosphate, 1 mM Na_3_VO_4_, 1 mg/ml aprotinin leupeptin and pepstatin and 1 mM phenylmethylsulfonyl fluoride] and centrifuged at 1,600 × g for 15 min at 4°C. A bicinchoninic acid (BCA) protein assay (Beyotime Institute of Biotechnology, Haimen, China) was utilized to measure the protein concentration in the supernatant. Equal amounts of protein were used for western blot analysis, which was performed with the following antibodies: Caspase-3 (#9661; 1:1000), Bcl-2 (#2870; 1:1,000) and BAX (#2772; 1:1000; all from Cell Signaling Technology, Inc.), and β-actin (#sc-47778; 1:1000; Santa Cruz Biotechnology, Inc., Dallas, TX, USA). The membrane was incubated with a horseradish peroxidase-conjugated secondary antibody for 1 h at 37°C. Blots were developed using an enhanced chemiluminescence kit (Pierce Biotechnology, Inc. Rockford, IL, USA).

### Statistical analysis

Data are presented as the mean ± standard error of the mean. SPSS software version 22 (SPSS, Inc., Chicago, IL, USA) was used for the statistical analysis to perform one-way analysis of variance, where P<0.05 was considered to indicate a statistically significant difference.

## Results

### Rutin prevents metabolic abnormalities

Hematological analysis revealed the metabolic characteristics of the experimental animals (Table I). In the DM group, STZ-induced diabetic rats exhibited a markedly lower body weight and higher blood glucose levels when compared with the control group (P<0.05). In addition, the heart-to-body weight ratio (HW/BW) in the DM group was significantly (P<0.05) higher compared with the control and rutin-treated groups, respectively (Table 1). Furthermore, rutin was shown to significantly decrease the blood glucose levels (to similar values to the control group) in the diabetic rats (P<0.05). In the basal fasting state, the DM group exhibited significantly (P<0.05) higher levels of TG and TC when compared with the control group. However, the level of TG was decreased in the DM + rutin group compared to the DM group. No statistically significant difference in the TC level was observed between the DM and DM + rutin groups.

### Rutin inhibits myocardial injury and oxidative stress

**Figure 1:**
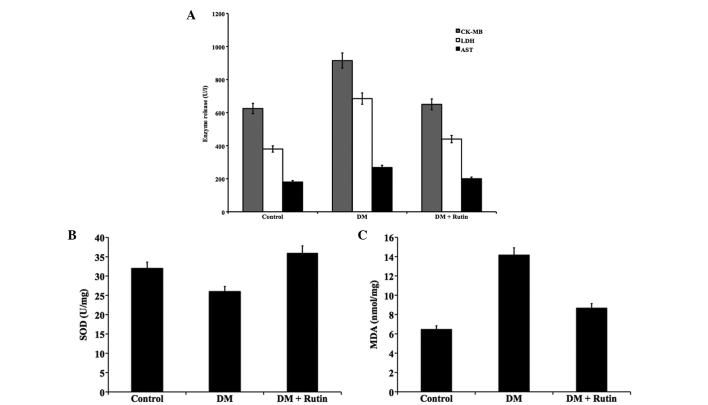
Rutin prevents myocardial injury and oxidative stress in an experimental model of DM in rats. Rutin was shown to (A) inhibit serum myocardial enzyme release, (B) increase SOD activity and (C) decrease the MDA content in the heart tissue. MDA, malondialdehyde; CK-MB, creatine kinase-MB; LDH, lactate dehydrogenase; AST, aspartate aminotransferase; SOD, superoxide dismutase; DM, diabetes mellitus.

The myocardial enzymes, CK-MB, LDH and AST, can be used as biochemical indicators of myocardial injury (Fig. 1A). When compared with the control group, the levels of the three enzymes were significantly increased in the DM group (P<0.05). In the rutin-treated DM group, decreased levels of myocardial enzymes were observed (P<0.05, vs. DM group); thus, rutin was shown to protect the diabetic rats against cardiac injury.

In the heart tissue samples of the DM group rats, a decrease in the activity of SOD (Fig. 1B) and an increase in the accumulation of lipid peroxides with a concordant increase in MDA content (Fig. 1C) were observed (P<0.05, vs. control group). Following treatment with rutin in the diabetic rats, the activity of SOD was found to be upregulated, while the MDA content was markedly decreased (P<0.05, vs. DM group).

### Rutin prevents the production of inflammatory factors

**Figure 2:**
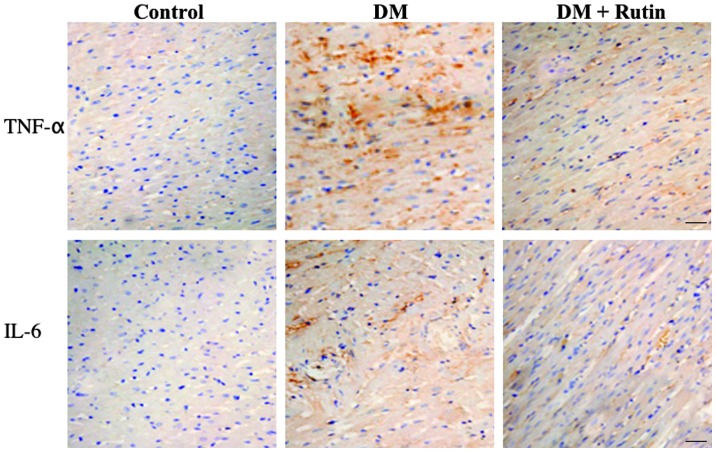
Immunohistochemical staining for myocardial TNF-α and IL-1β expression. Brown staining indicates the cells with positive expression (scale bar, 50 mm; magnification, ×20). DM, diabetes mellitus; TNF, tumor necrosis factor; IL, interleukin.

Immunohistochemical analysis revealed increased staining for the inflammatory factors, TNF-α and IL-6, in the DM group when compared with the control group. However, decreased levels of staining (TNF-α and IL-6) were observed in the rutin-treated group when compared with the DM group (Fig. 2). These observations indicate the protective effect of rutin against inflammation.

### Rutin inhibits DM-induced apoptosis of cardiomyocytes

**Figure 3:**
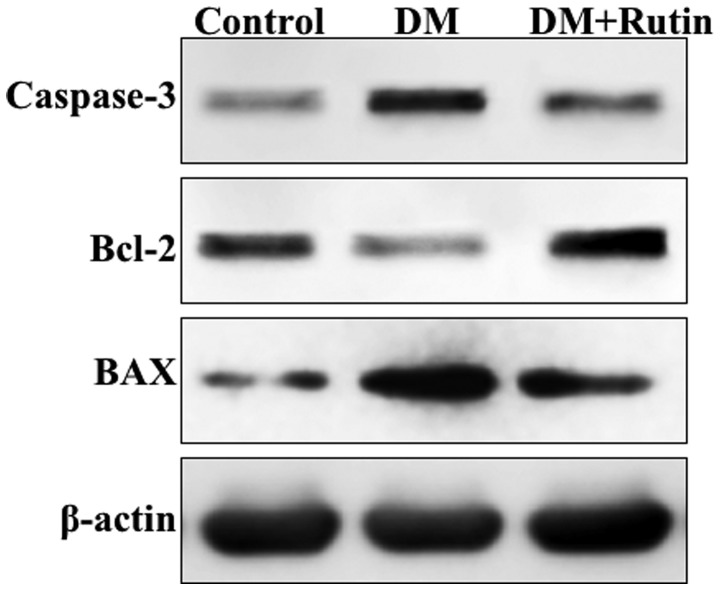
Western blot analysis showing the protein expression levels of caspase-3, Bcl-2 and BAX. β-actin was used as a control. DM, diabetes mellitus.

The expression levels of the antiapoptotic protein, Bcl-2, and proapoptotic proteins, BAX and caspase-3, were assessed by immunoblotting. The blots revealed enhanced expression levels of caspase-3 and BAX, but a reduced expression of Bcl-2 in the DM group when compared with the control group. Notably, the diabetic rats treated with rutin exhibited significantly increased protein expression levels of Bcl-2, and downregulated protein expression levels of caspase-3 and BAX (Fig. 3).

## Discussion

The metabolic abnormalities observed in the DM group, including the markedly higher concentrations of plasma and serum glucose (P<0.05), were the result of insulin secretion inhibition caused by the ROS produced by STZ. The ROS subsequently repressed the function of the antioxidant system, while causing oxidative damage to the pancreatic β-cells (19). Although the concentration levels of plasma and serum glucose in the DM + rutin group were not reduced to the same extent as that observed in the control group, the levels were significantly decreased compared with the level in the DM group (P<0.05). As shown in the study by Kamalakkannan and Prince (20), the concentration levels of plasma glucose and serum glucose decreased as rutin removed free radicals and repressed lipid peroxidation, while protecting the β-cells by impeding the oxidative stress caused by STZ and increasing the level of insulin secretion. The different forms of DM include type 1 (insulin-dependent), type 2 (non-insulin-dependent) and gestational diabetes. Using a low dose of STZ combined with a high-energy intake is considered to be a general strategy to obtain an animal model of type 2 DM, since these factors simulate the real course of the human disease (21,22). In the present study, STZ injections were shown to be successful in inducing DM by markedly elevating the levels of serum glucose, TG and TC. Through using this method, the serum symptoms exhibited an increased similarity to those of type 2 DM compared with those of type 1 DM.

DCM is classified as ventricular dysfunction with an increased risk of cardiac failure, in the absence of hypertension, coronary artery and valvular heart diseases (23). The condition is frequently observed in humans and animals. Consistent with a previous study (24), the untreated DM rats in the present study were characterized by a decreased or attenuated antioxidant defense, as shown by the decreased SOD activity, accompanied with increased myocardial lipid peroxidation and inactivation of prosurvival pathways of Bcl-2, eventually culminating in cell apoptosis and increased levels of inflammation. By contrast, the administration of rutin was demonstrated to prevent the development of these characteristic alterations of DCM. The beneficial effects of rutin may be explained in part as follows. Firstly, rutin treatment was shown to decrease the elevated levels of blood glucose and TG. A previous study demonstrated that the onset of cardiovascular complications may be delayed by controlling metabolic abnormalities (25).

Secondly, rutin was found to attenuate oxidative stress. The compound has previously been demonstrated to intercept and neutralize ROS using its potential antioxidant function (17). Oxidative stress is defined as an imbalance between the production and elimination of free radicals, which play a critical role in the development of heart failure and left ventricular remodeling in DCM (26). Hyperglycemia has been shown to exacerbate glucose oxidation and the generation of ROS in the mitochondria (27), which subsequently results in DNA damage and an accelerated rate of apoptosis. NADPH oxidase is a critical determinant of myocardial ROS generation (28). In the present study, rutin was demonstrated to decrease the level of SOD activity and reduce lipid peroxidation in a rat model of DM.

Thirdly, rutin was shown to suppress cardiac inflammation, which is characterized by increased levels of proinflammatory cytokines. Proinflammatory cytokines, including IL-6 and TNF-α, are critical in the manifestation of DCM (29). Rutin has a number of properties, including antioxidant activities, anti-inflammatory effects (14), myocardial protection (15) and hepatoprotective activities (16), that enable the suppression of cardiac inflammation. Rutin is hypothesized to exert protective effects for various organs in DCM rats through dynamic medical functions. In addition, according to a previous study, antioxidants impede inflammation (30), and it is known that the antioxidative activity and anti-inflammatory effects of rutin are not an independent function.

In conclusion, the results demonstrated that rutin may have great therapeutic potential in the treatment of DCM, and possibly other cardiovascular disorders, by ameliorating metabolic abnormalities, oxidative stress, inflammation and cellular apoptosis pathways.

## Figures and Tables

**Table I:** Rutin prevents metabolic abnormalities.

Group	Body weight (g)	HW/BW (mg/g)	Blood glucose (mmol/l)	TG (mmol/l)	TC (mmol/l)
Control	415±16	2.77±0.16	5.3±0.3	0.79±0.07	1.26±0.07
DM	257±17^a^	4.25±0.18^a^	21.5±1.2^a^	1.24±0.11^a^	1.51±0.09^a^
DM + rutin	345±21	3.32±0.19	9.2±1.9^b^	0.93±0.04	1.35±0.12

Body weight and heart weight were measured on the day that the rats were sacrificed. Blood glucose, TG and TC levels were measured in the basal fasting state on the day that the rats were sacrificed. Data are expressed as the mean ± standard error of the mean;

a P<0.05, vs. control group;

b P<0.05, vs. DM group (n=8 per group).

TG, triglycerides; TC, total cholesterol; HW/BW, heart-to-body weight ratio; DM, diabetes mellitus.
